# Case Report: Molecular and immunological insights into primary extramedullary plasmacytoma: discovery of a novel *IGH::NFKB1* fusion and its impact on disease progression and treatment

**DOI:** 10.3389/fimmu.2025.1664103

**Published:** 2025-10-22

**Authors:** Ziting Gao, Dongbing Li, Tingting Zhang, Wenfeng Su, Jintao Xu, Yuanjie Zhuang, Rong Cao, Yufei Xie, Xingping Lang, Huafei Chen, Chunlin Fan, Xi Yang, Hongming Huang, Dan Guo

**Affiliations:** ^1^ Department of Hematology, Affiliated Hospital of Nantong University, Medical School of Nantong University, Nantong, Jiangsu, China; ^2^ Molecular Genetics Laboratory, Advanced Molecular Pathology Institute of Soochow University and SANO, Suzhou, Jiangsu, China; ^3^ Molecular Genetics Laboratory, Suzhou Sano Precision Medicine Ltd., Suzhou, Jiangsu, China; ^4^ Department of Hematology, Affiliated Hospital of Nantong University, Nantong, Jiangsu, China

**Keywords:** extramedullary plasmacytoma, bone destruction, IGH::NFKB1 fusion, NF-κB pathway, chemotherapy, CAR-T therapy

## Abstract

Extramedullary Plasmacytoma (EMP) is a rare plasma cell neoplasm that originates outside the bone marrow. Primary Extramedullary Plasmacytoma with Diffuse Lymph Node Involvement (PLNEMP) is exceptionally rare. Here, we report a unique case of PLNEMP and significant bone destruction, characterized by a novel *IGH::NFKB1* fusion gene. A 60-year-old Chinese male presented with palpable enlarged lymph nodes in the left inguinal region. After completing laboratory tests and examinations, it was suggested that there was monoclonal immunoglobulinemia and multiple bone destruction. Pathological examination of the left inguinal lymph node biopsy showed plasmacytoma with monoclonal gammopathy. Genomic profiling identified a novel *IGH::NFKB1* fusion gene. The two 3′ regulatory region (3′RR) enhancers of the *IGH* locus were fused to a region 379 bp upstream of *NFKB1* exon 1, resulting in overexpression of *NFKB1*. The patient received four cycles of chemotherapy with Mitoxantrone hydrochloride liposome (Lipo-MIT) combined with Bortezomib, Pomalidomide, and Dexamethasone (MVPD), achieving very good partial remission (VGPR) in hematological and partial remission (PR) in extramedullary disease. Subsequently, he underwent autologous stem cell transplantation (ASCT) followed by BCMA CAR-T cell therapy. At 8 months post-transplantation, complete remission (CR) was achieved in hematological parameters, and the extramedullary disease showed a response greater than PR. The patient has survived for 26 months so far. This case highlights the importance of recognizing the rare presentation of PEMP with diffuse lymph node involvement and significant bone destruction. The presence of the novel *IGH::NFKB1* fusion gene provides insights into the potential role of the NF-κB pathway in the pathogenesis of this disease. The successful treatment with MVPD chemotherapy, ASCT, and BCMA CAR-T therapy demonstrates the potential efficacy of this combined therapeutic approach in achieving long-term remission and survival in such rare cases. Further studies are warranted to explore the therapeutic implications of targeting the NF-κB pathway in similar cases of EMP with bone destruction.

## 1 Introduction

Extramedullary plasmacytoma (EMP) is a rare plasma cell neoplasm that originates outside the bone marrow, accounting for approximately 3-5% of all plasma cell malignancies and having an incidence rate of 0.09 per 100,000 individuals (1). EMP can be categorized into primary and secondary types based on its occurrence in relation to multiple myeloma (MM). Primary extramedullary plasmacytoma (PEMP) typically exhibits lower aggressiveness compared to secondary EMP and rarely progresses to MM. PEMP usually presents as a solitary tumor in soft tissues, most frequently in the upper respiratory tract, including the nasal cavity, paranasal sinuses, and nasopharynx (2). However, extramedullary plasmacytoma with lymph node involvement (PLNEMP) is an exceptionally rare manifestation, with only a few cases reported worldwide (3–9). The diagnosis of PLNEMP is challenging due to the overlap of its morphology and immunophenotype with other lymphoid malignancies.

The NF-κB signaling pathway plays a crucial role in the pathogenesis of MM. This pathway regulates osteoclast formation, activation, and survival during normal bone remodeling through the *RANKL*/*RANK*/*OPG* axis (10). Aberrant activation of NF-κB can enhance the secretion of matrix metalloproteinases (MMPs), bone morphogenetic proteins (BMPs), and insulin-like growth factor 1 (IGF-1) by osteoclasts, thereby promoting bone destruction as well as the survival and proliferation of MM cells (11, 12). Genetic alterations involving the NF-κB pathway have been identified in various plasma cell disorders, including EMP and MM.

In this report, we present a unique case of primary extramedullary plasmacytoma characterized by diffuse lymph node involvement and significant bone destruction. Molecular pathological testing revealed a novel *IGH::NFKB1* fusion gene, which may contribute to the marked bone destruction observed in this patient. We discuss the potential role of this fusion gene in the context of the NF-κB signaling pathway and its implications for the pathogenesis of EMP. Additionally, we review the clinical presentation, diagnostic challenges, and therapeutic approaches for this rare condition, highlighting the importance of recognizing and targeting the NF-κB pathway in the management of such cases.

## 2 Case presentation

### 2.1 Clinical findings

A 60-year-old Chinese male presented with palpable enlarged lymph nodes in the left inguinal region, which had gradually increased in size over three years. The patient sought medical attention on June 6, 2023, due to the progressive enlargement of the lymph nodes. Immunofixation electrophoresis revealed an abnormal concentration of the aggregated band of IgG Kappa light chains. The serum-free light chain levels were Kappa: 7800 mg/dL and Lambda: 278 mg/dL, with no abnormalities detected in urine light chains. The free light chain (FLC) results indicated a Kappa level of 53.42 mg/dL and a Lambda level of 20.07 mg/dL, yielding a ratio of 2.66. Serum protein electrophoresis demonstrated a total protein level of 112.5 g/L, with M protein constituting 58.275 g/L. The IgG level was measured at 83.80 g/L. Additional blood tests indicated that levels of red blood cells, hemoglobin, white blood cells, and platelets were within normal ranges. Albumin (ALB) was recorded at 29 g/L, and β2-microglobulin (β2-MG) was 4.02 mg/L. Furthermore, blood urea nitrogen (BUN), creatinine, blood calcium, aspartate aminotransferase (AST), alanine aminotransferase (ALT), lactate dehydrogenase (LDH), and serum electrolyte levels all showed no significant abnormalities. The patient has a history of acute hepatitis infection from decades ago, which resulted in positive HBV-related antibodies (anti-HBs, anti-HBe, and anti-HBc).

### 2.2 Gross examination, histology and immunohistochemical study

**Figure 1:**
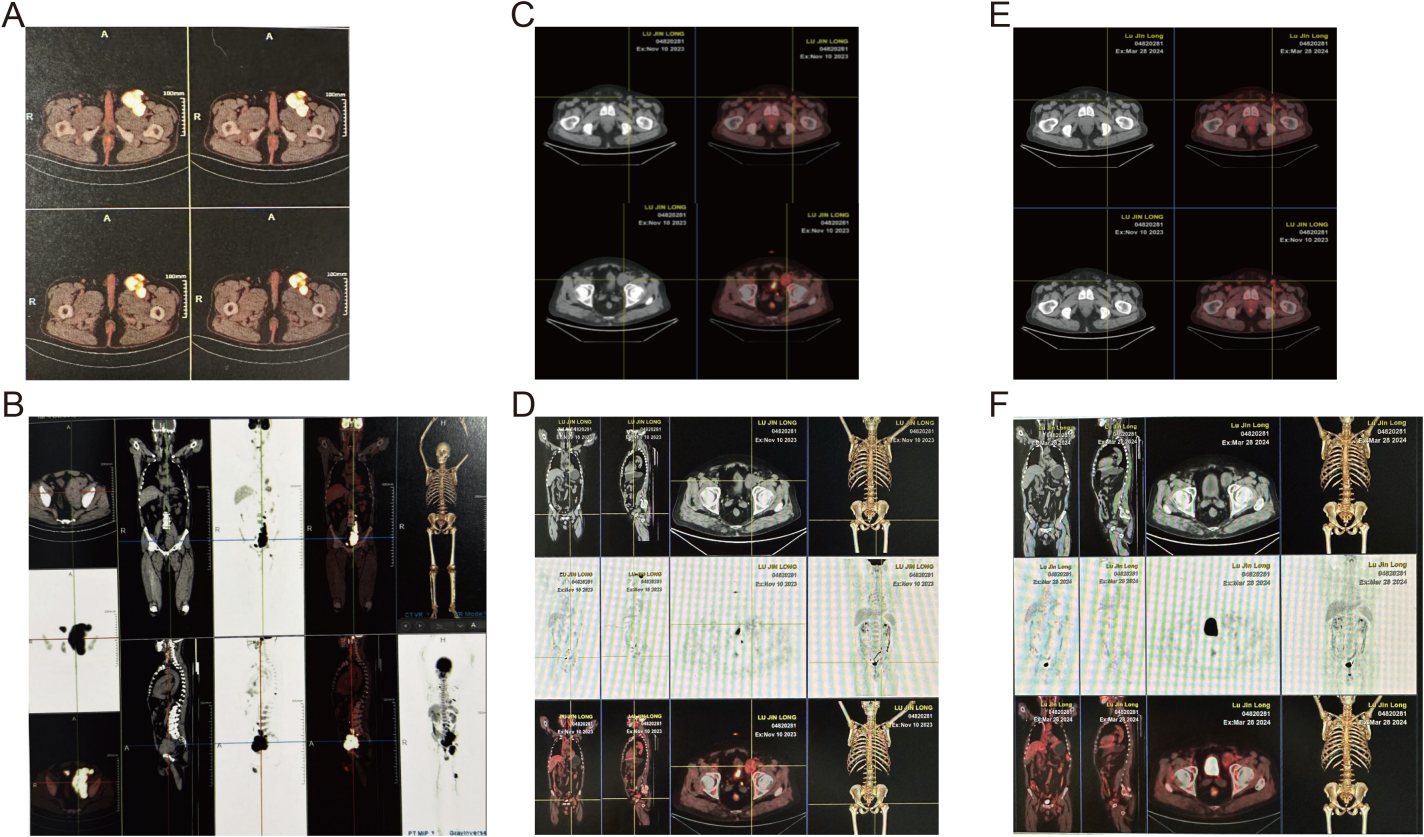
Whole-body ^18^F-FDG PET-CT assessment of disease extent and sequential therapeutic response. **(A, B)** Pre-treatment MIP **(A)** and matching axial pelvis image **(B)** showing widespread hyper-metabolic lymphadenopathy (SUVmax 15.9) and osteolytic destruction of the left ilium (SUVmax 14.6). **(C, D)** After four cycles of MVPD regimen the MIP **(C)** and axial pelvis image **(D)** reveal marked reduction in nodal size and metabolic activity (residual SUVmax 5.2; iliac lesion SUVmax 4.4). **(E, F)** One-month post-autologous stem-cell transplantation plus BCMA CAR-T infusion the MIP **(E)** and axial pelvis image **(F)** demonstrate complete metabolic resolution with no detectable abnormal uptake, consistent with hematologic complete remission and extra-medullary response > partial remission.

Imaging studies, including plain abdominal and pelvic CT scans, revealed multiple space-occupying lesions in the left pelvic cavity, left inguinal region, and retroperitoneum, suggestive of enlarged lymph nodes with partial fusion. Significant bone destruction was observed in the left ilium. Positron emission computed tomography (PET-CT) demonstrated increased metabolic activity in multiple ribs, the sternum, the left ilium, and the hip joint, with a standardized maximum uptake value (SUVmax) of 14.6. Multiple enlarged and partially fused lymph nodes were identified in various anatomical regions, with the largest lymph node in the left inguinal region measuring 44 x 45 mm and exhibiting a SUVmax of 15.9 (**Figures 1A, B**).

**Figure 2:**
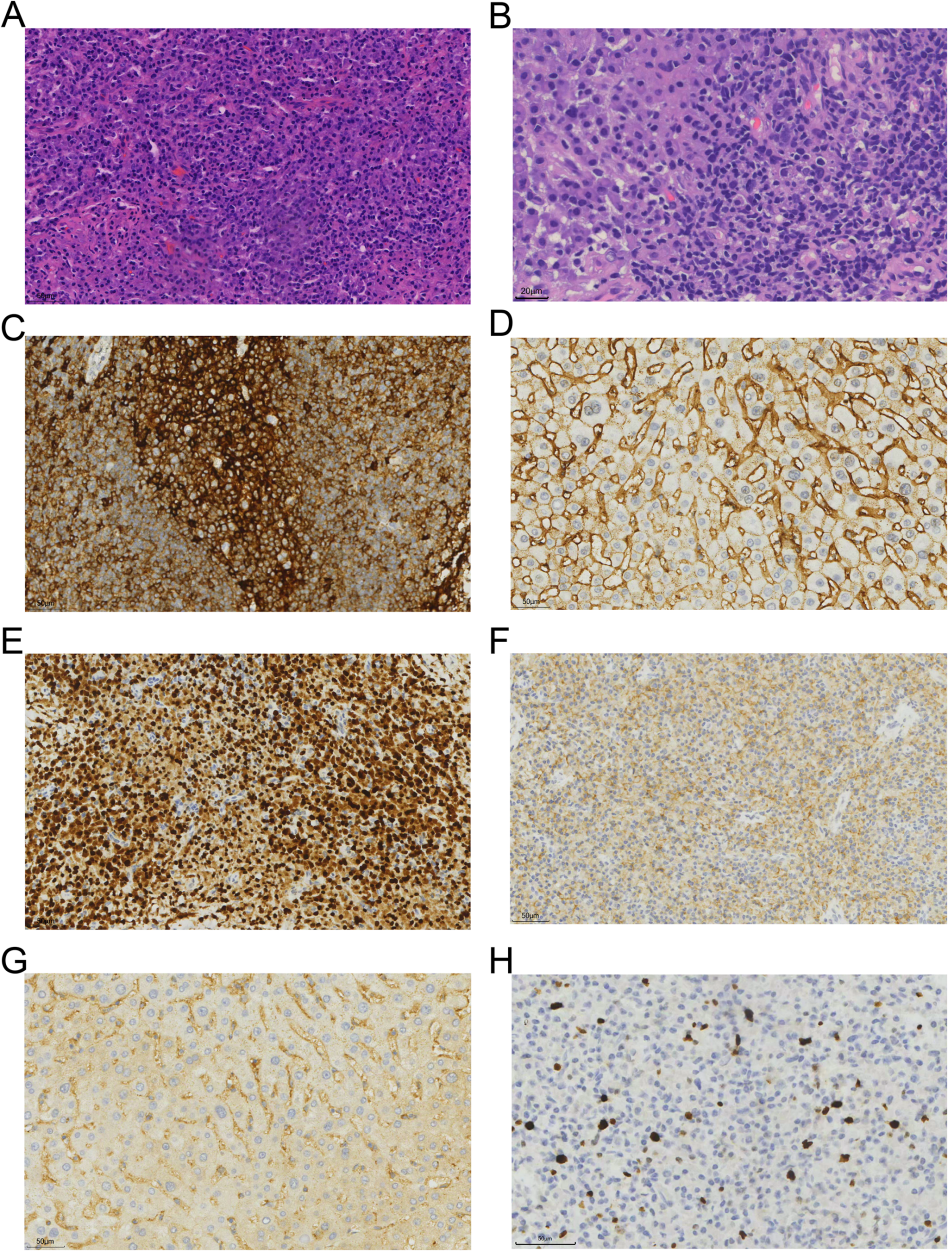
Histopathological and immunohistochemical features of the tumor. **(A, B)** Hematoxylin and eosin (H&E) staining of the left inguinal lymph node biopsy, showing a dense infiltration of plasma cells with eccentric nuclei and abundant cytoplasm (original magnifications, ×40 and ×80, respectively). **(C–H)** Immunohistochemical staining was positive for CD38 (original magnification, ×40; 1C), CD138 (1D), MUM-1 (1E), CD117 (1F), Igκ (1G), and Ki67 (5%, 1H).

A tissue biopsy was performed on the left inguinal lymph node. Histopathological examination revealed the presence of plasma cells characterized by CD38+, CD138+, MUM-1+, CD117+, and Igκ-restricted expression, consistent with plasmacytoma (**Figure 2**). The Ki67 positivity rate was approximately 5%. The low Ki67 expression rate and normal levels of lactate dehydrogenase (LDH) suggested a relatively indolent course, consistent with the characteristics of diffuse lymph node involvement in primary extramedullary plasmacytoma.

### 2.3 Molecular analysis

**Figure 3:**
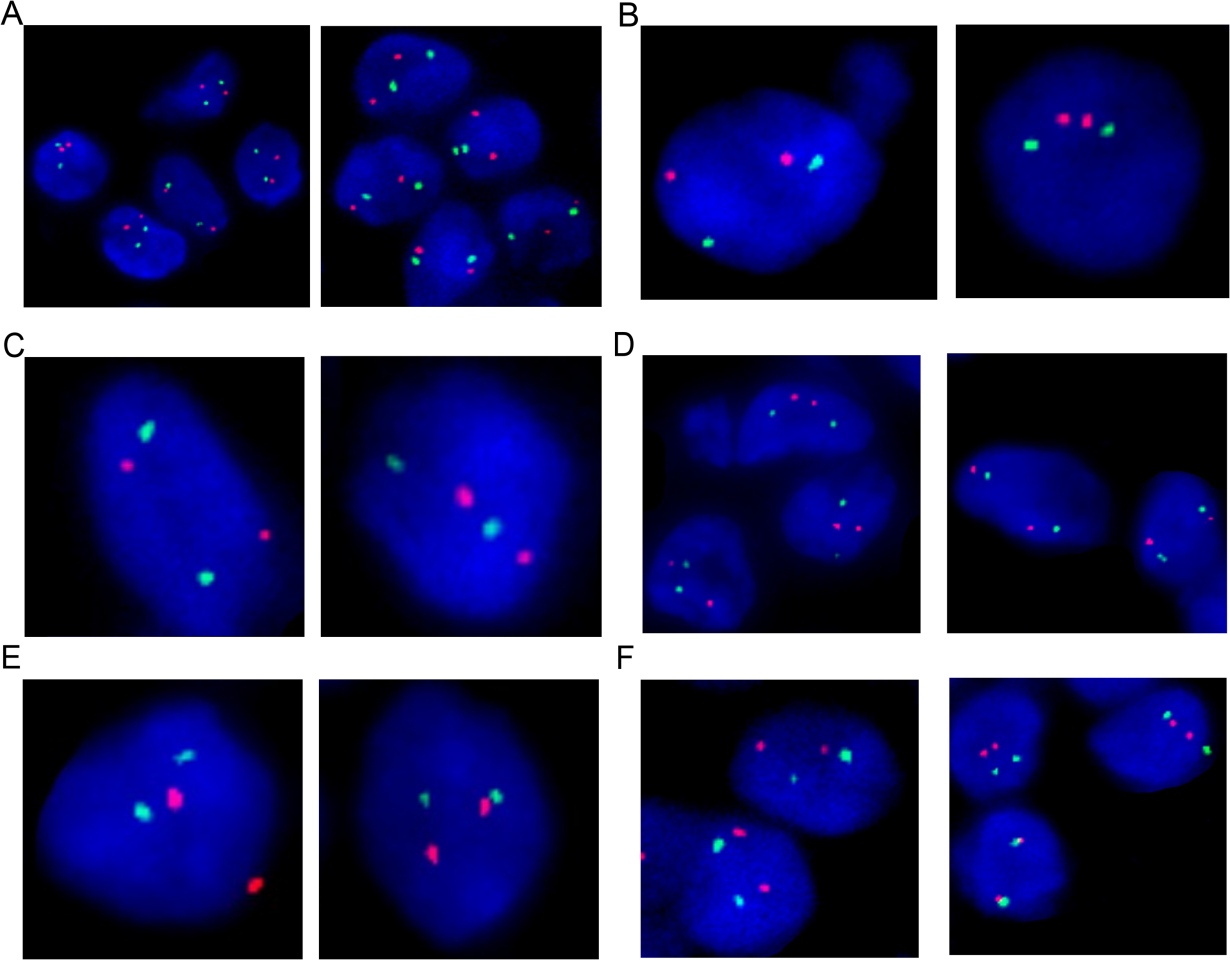
Fluorescence In Situ Hybridization (FISH) analysis of the lymph node biopsy showed no aberrations on 1p32/1q21 **(A)**, IGH/FGFR3 **(B)**, IGH/CCND1 **(C)**, 13q **(D)**, IGH/MAF **(E)**, and P53 **(F)**.

**Figure 4:**
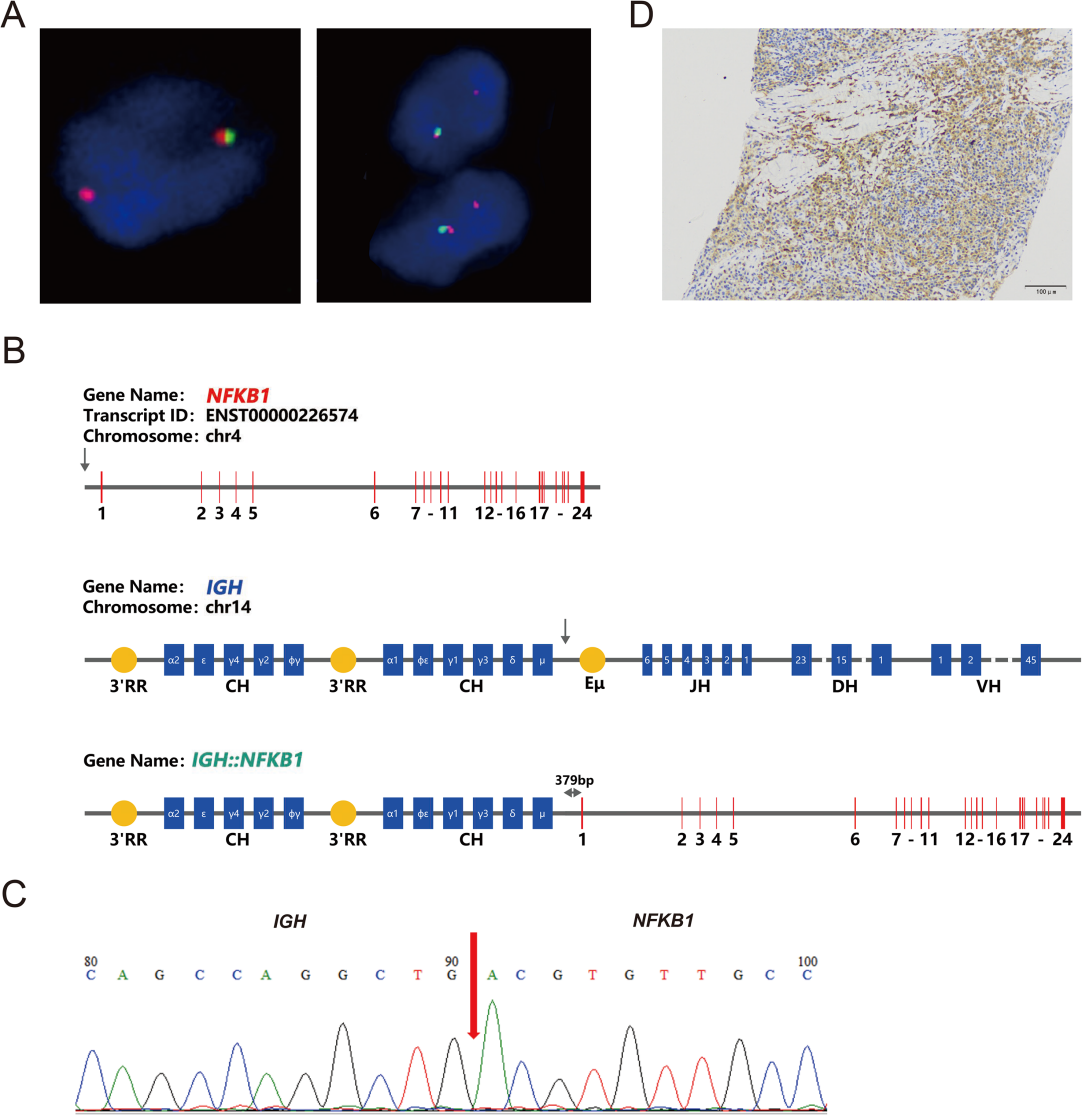
Molecular characteristics of the *IGH*::*NFKB1* fusion gene. **(A)** Fluorescence *In Situ* Hybridization (FISH) analysis of *IGH* on the lymph node biopsy showed loss of the 5’IGH region, consistent with an unbalanced *IGH* rearrangement. **(B)** Schematic representation of the *IGH*::*NFKB1* fusion gene. The arrows indicate the breakpoints of *IGH* and *NFKB1*. The *IGH*::*NFKB1* rearrangement places the *IGH* 3′RR 379 bp upstream of exon 1 of the *NFKB1* gene. 3′RR, 3′ Regulatory Region; CH, Constant region; JH, Joining region; DH, Diversity region; VH, Variable region; Eμ, enhancer. **(C)** PCR amplification of the *IGH*::*NFKB1* fusion followed by Sanger sequencing confirmed the rearrangement. **(D)** Immunohistochemical staining was positive for *NFKB1* expression (original magnification, ×20).

Fluorescence *in situ* hybridization (FISH) analysis was conducted using the MM FISH probe set, which included probes for 1p32/1q21, FGFR3/IGH, CCND1/IGH, 13q, IGH, MAF/IGH, and P53 (**Figure 3**). Interphase FISH was performed on 4-μm formalin-fixed paraffin-embedded (FFPE) lymph-node sections using the following commercially available probes (HealthCare, Wuhan, China): 1p32/1q21 (FP-197), 13q14 deletion (FP-025), TP53 (17p13) deletion (FP-014-2), IGH break-apart (FP-242-3), IGH/CCND1 dual-fusion (FP-233-1), IGH/MAF dual-fusion (FP-233-2), IGH/MAFB dual-fusion (FP-233-3), and IGH/FGFR3 (FP-233-4). Hybridization, post-wash and counter-staining were carried out according to the manufacturer’s instructions. For each probe, ≥100 interphase nuclei were scored; cut-off values for positivity were set at 10% for split-signal probes and 15% for deletion probes. The results showed partial deletion or imbalanced rearrangement at the 5’ end of the *IGH* gene in approximately 57% of cells (**Figure 4A**), but no common gene fusions with *FGFR3*, *CCND1*, or *MAF* were detected. Comprehensive molecular profiling of 30 MM-related genes, including immunoglobulin genes *IGH*, *IGL*, and *IGK*, identified a novel *IGH* non-classical fusion gene. The two 3′ regulatory region (3′RR) enhancers of the *IGH* locus were fused to a region located 379 bp upstream of *NFKB1* exon 1 (**Figure 4B**). This fusion was further characterized using a PCR assay with *IGH*- and *NFKB1*-specific primers (*IGH*-F1: GGTCAGCTTAGGTCAGTTTTGC; nested *IGH*-F2: TGAGTCCATTTCTGAAAGCTGG; *NFKB1*-R1: GAAGCCCGCACTTCTAGGG; nested *NFKB1*-R2: GGCTCTGGCTTCCTAGCAG). Sanger sequencing of the PCR product confirmed the presence of the *IGH*::*NFKB1* rearrangement (**Figure 4C**). This structural alteration juxtaposes the potent IGH enhancers directly upstream of NFKB1, potentially driving its ectopic expression. To assess the functional consequence of this rearrangement, Immunohistochemical (IHC) was performed and demonstrated strong NFKB1 expression in the tumor specimen (**Figure 4D**).

IHC analysis revealed nuclear positivity for phospho-p65 (Ser536) in several tumor cells, indicating activation of the canonical NF-κB pathway (**Supplementary Figure S1A**). In contrast, staining for p100 showed predominant cytoplasmic localization without significant nuclear accumulation of p52, suggesting that the alternative NF-κB pathway was not substantially activated in this case (**Supplementary Figure S1B**). These results demonstrate that the *IGH::NFKB1* fusion preferentially activates the canonical NF-κB signaling cascade, while the alternative pathway remains inactive.

### 2.4 Treatment and outcome

For patients with extramedullary plasmacytoma (EMP), incorporate chemotherapy agents into treatment regimens are recommended to control disease progression. Refer to a registry clinical study conducted by our center (Mitoxantrone Hydrochloride Liposome Combination Regimen in the Treatment of High-risk/Extramedullary Multiple Myeloma, Clinical Trial Number: CTR20212318), the patient underwent four cycles of chemotherapy with a regimen consisting of liposomal mitoxantrone hydrochloride (Lipo-MIT, 10 mg on days 1 and 15), bortezomib (2.57 mg per week), pomalidomide (4 mg from day 1 to day 14), and dexamethasone (20 mg per week) (MVPD). Targeted at bone destruction, denosumab injection 120 mg was administered once monthly to inhibit bone destruction. Regular follow-up evaluations included immunofixation electrophoresis, blood and urine light chain tests, and imaging studies to monitor disease status. M protein decreased from 58.275 g/L after the first cycle to 40.110 g/L, and maintained a minimal amount after 4th cycle treatment.

The patient achieved very good partial remission (VGPR) in hematological efficacy and PR in extramedullary disease. Subsequently, the patient underwent autologous stem cell transplantation (Auto-ASCT) followed by BCMA CAR-T cell therapy. PET/CT was performed before treatment, after 4 cycles of treatment and after Auto-ASCT, showing that the volume and tumor activity of lymph nodes was reduced (the maximum was 7.5 cm → 4.0 cm → 3.4 cm, SUVmax: 19.2 → 5.2 → 3.4); the bone destruction was significantly allevi, SUVmax:14.6 → 4.4 → 3.4) (**Figures 1C, D**). At 1-month post-transplantation, complete remission (CR) was achieved in hematological parameters, and the extramedullary disease showed a response greater than PR (**Figures 1E, F**). Post-transplantation maintenance therapy was initiated with a combination of bortezomib and lenalidomide (VR). The patient has survived for 26 months so far, showing negative for M protein, and about a 6 * 7.5 mm lymph node in the left inguinal region.

This case highlights the importance of molecular diagnostics in identifying actionable genetic alterations and guiding personalized treatment strategies. The presence of the novel *IGH::NFKB1* fusion gene provides insights into the potential role of the NF-κB pathway in the pathogenesis of this disease. The successful treatment with MVPD chemotherapy, ASCT, and BCMA CAR-T therapy demonstrates the potential efficacy of this combined approach in achieving long-term remission and survival in such rare cases.

## 3 Discussion

PLNEMP is exceedingly rare—only nine cases have been reported worldwide (5, 7, 9)—and its presentation with prominent bone destruction while sparing the marrow creates immediate diagnostic confusion. International Myeloma Working Group (IMWG) criteria can be fulfilled on paper (biopsy-proven plasmacytoma plus ≥ 2 osteolytic lesions) (13), yet the patient presented with left inguinal lymphadenopathy 3 years before diagnosis and slowly enlarged to multiple lymph throughout the body, lacked the classical CRAB tetrad (anemia, hypercalcemia, renal impairment, bone pain), showed only 0.5% marrow plasma cells and a clonal burden of 0.01% by high-sensitivity flow, and exhibited an indolent tempo (Ki-67 5%, LDH consistently normal). These features argue against a diagnosis of *de-novo* multiple myeloma (MM) and favor PLNEMP that secondarily eroded bone. The patient’s multiple bone lesion may have resulted from the progression of extramedullary plasmacytoma, representing a biological course distinct from classical multiple myeloma. Plasmablastic lymphoma was excluded because the patient was HIV-negative, immunocompetent, lacked MYC rearrangement and displayed a low proliferation index (14). Other small B-cell lymphomas with plasmacytic differentiation (MALToma, follicular lymphoma, monomorphic B-cell lymphoma) were discounted on the absence of a background population of small neoplastic B cells and the uniform CD38+/CD138+/MUM-1+/Igκ-restricted plasma-cell phenotype (9). We therefore conclude that this is the first documented case of PLNEMP that evolved into “myeloma-like” bone destruction while retaining minimal marrow involvement, a trajectory underscored by the novel *IGH::NFKB*1 fusion identified by comprehensive molecular profiling.

IGH has fused with a variety of genes to form a number of different fusion forms. IGH fusions with oncogenes mainly include *IGH::MYC*, *IGH::BCL2*, *IGH::BCL6*, *IGH::CCND1*, *IGH::FGFR3*, *IGH::NSD2*, *IGH::MMSET*, *IGH::PAX5*, and *IGH::SOX9* (15, 16). IGH fusions with other genes mainly include *IGH::CRLF2*, *IGH::EPOR*, *IGH::IL3*, *IGH::MALT1*, *IGH::BACH2*, *IGH::FOXP1*, and *IGH::IRF4* (17). These IGH fusion forms are pathologically important in different malignant tumors, affecting disease diagnosis, prognosis, and treatment strategies. No NFKB1 fusion genes have been reported so far.

This report presents a rare case of PLNEMP with significant bone destruction, accompanied by a novel *IGH::NFKB1* fusion gene. Although genome-wide surveys have detected NFKB1 rearrangements in myeloma (e.g. *PIM2::NFKB1*) (18) and have exhaustively catalogued IGH translocation partners (19), an *IGH::NFKB1* fusion has never been reported, supporting the novelty of the alteration described here. This case expands the clinical and molecular spectrum of EMP and highlights the importance of comprehensive molecular diagnostics in identifying unique genetic alterations that may guide targeted therapies.

Unlike previous reports, our patient exhibited significant bone destruction and minimal bone marrow involvement, which are atypical features for PLNEMP. Molecular analysis revealed a novel *IGH::NFKB1* fusion gene, characterized by high expression level of NFKB1 influenced by the IGH enhancer. This genetic alteration may contribute to the pronounced bone destruction observed in this patient by enhancing NF-κB activity and promoting the secretion of matrix metalloproteinases, bone morphogenetic proteins, and insulin-like growth factor 1 through the RANKL/RANK/OPG pathway (10–12).

The treatment of EMP, especially PLNEMP, remains challenging due to its rarity and complex clinical manifestations. Previous reports on PLNEMP have shown variable responses to different treatment modalities. Systemic chemotherapy, particularly regimens incorporating bortezomib, has demonstrated promising results, with some patients achieving near CR (3–9). For example, a patient underwent 4 cycles of vincristine, doxorubicin and dexamethasone (VAD) therapy followed by Auto-ASCT, however, relapse still occurred after a brief PR. Eventually, the patient achieved complete remission and remained quite a long time stable with 8 cycles of bortezomib (14). Notably, radiotherapy alone has shown limited efficacy in some cases (7). In our case, the patient presented with extensive lymph node involvement at diagnosis, precluding surgical resection. The patient also exhibited significant bone destruction, intramedullary micro-infiltration and M-protein secretion. Consequently, we employed a combination of bortezomib and cytotoxic agents for systemic chemotherapy, followed by ASCT and BCMA CAR-T cell therapy. This multimodal approach resulted in excellent clinical outcomes, with CR achieved in hematological parameters and a response greater than PR in extramedullary disease. The patient has now survived for 26 months, highlighting the potential efficacy of this treatment strategy. Our case supports the use of a combination approach, including ASCT and CAR-T therapy, as a potentially effective strategy for managing PLNEMP with significant bone destruction.

The identification of the novel *IGH::NFKB1* fusion gene in this case underscores the importance of molecular diagnostics in uncovering actionable genetic alterations in rare plasma cell neoplasms. Further studies are warranted to elucidate the biological functions of this fusion gene and its role in disease progression. Although *NFKB1* rearrangement theoretically could affect both pathways, our IHC data support selective canonical NF-κB activation in this patient; whether micro-environmental cues or additional hits are required to unleash the alternative axis warrants further investigation. Additionally, large-scale genomic studies are needed to determine the prevalence of such genetic alterations in other EMP cases and related plasma cell disorders. The development of targeted therapies against specific genetic drivers, such as NF-κB1, may offer new therapeutic opportunities for patients with aggressive forms of EMP.

## 4 Conclusion

In conclusion, this case highlights the clinical and molecular complexity of PLNEMP. The successful treatment outcome achieved through a multimodal approach, including chemotherapy, ASCT, and CAR-T therapy, underscores the potential benefits of personalized treatment strategies guided by comprehensive molecular diagnostics. Future research should focus on exploring the biological significance of novel genetic alterations and developing targeted therapies to improve patient outcomes in rare plasma cell neoplasms.

## Data Availability

The original contributions presented in the study are included in the article/**Supplementary Material**. Further inquiries can be directed to the corresponding authors.

## Supplementary material

Supplementary Figure 1 Functional validation of NF-κB pathway activation status driven by the *IGH::NFKB1* fusion. **(A)** Phospho-p65 (Ser536) immunohistochemistry reveals weak nuclear staining, confirming constitutive activation of the canonical NF-κB pathway in tumor cells. Original magnification, ×40. **(B)** Staining for p100 (precursor of p52) demonstrates its cytoplasmic retention and absence of nuclear p52, suggesting that the alternative NF-κB pathway is not activated in this case. Original magnification, ×20.
