# Supplementary figures and images for: Case Report: Molecular and immunological insights into primary extramedullary plasmacytoma: discovery of a novel IGH::NFKB1 fusion and its impact on disease progression and treatment
Source: Front Immunol. 2025 Oct 22;16:1664103. doi: 10.3389/fimmu.2025.1664103 (PMC12585950; doi:10.3389/fimmu.2025.1664103)

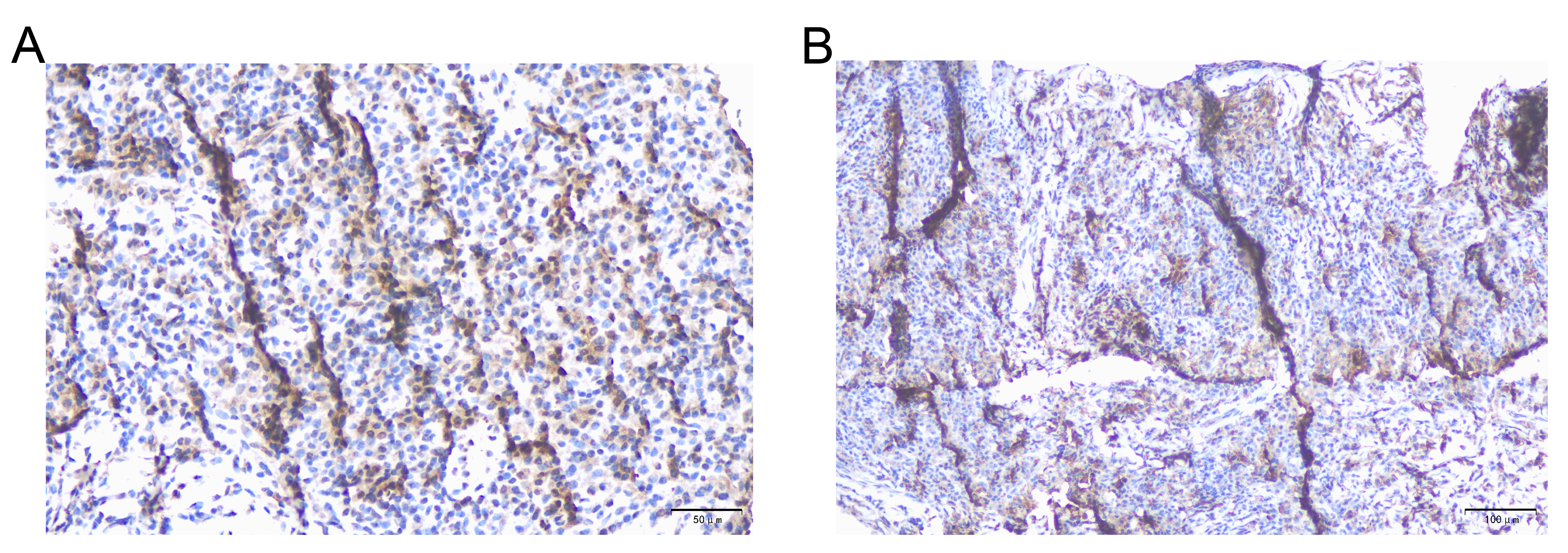

Supplement: Supplementary Figure 1 — Functional validation of NF-κB pathway activation status driven by the IGH::NFKB1 fusion. (A) Phospho-p65 (Ser536) immunohistochemistry reveals weak nuclear staining, confirming constitutive activation of the canonical NF-κB pathway in tumor cells. Original magnification, ×40. (B) Staining for p100 (precursor of p52) demonstrates its cytoplasmic retention and absence of nuclear p52, suggesting that the alternative NF-κB pathway is not activated in this case. Original magnification, ×20. [file Image1.tif]
